# Effect of Glycemic Index of Breakfast on Energy Intake at Subsequent Meal among Healthy People: A Meta-Analysis

**DOI:** 10.3390/nu8010037

**Published:** 2016-01-04

**Authors:** Feng-Hua Sun, Chunxiao Li, Yan-Jie Zhang, Stephen Heung-Sang Wong, Lin Wang

**Affiliations:** 1 Department of Health and Physical Education, Hong Kong Institute of Education, 10 Lo Ping Road, Tai Po, Hong Kong, China; fhsun@ied.edu.hk (F.-H.S.); cxli@ied.edu.hk (C.L.); 2 School of Humanities and Social Science, Chinese University of Hong Kong, Shenzhen 2001, China; zhangyanjie@cuhk.edu.cn; 3 Department of Sports Science and Physical Education, Chinese University of Hong Kong, Shatin, Hong Kong, China; hsswong@cuhk.edu.hk; 4 Key Laboratory of Exercise and Health Science of the Ministry of Education, Shanghai University of Sport, Shanghai 200438, China

**Keywords:** low glycemic index, appetite, meta-analysis

## Abstract

Meals with low glycemic index (GI) may suppress short-term appetite and reduce subsequent food intake compared with high-GI meals. However, no meta-analysis has been conducted to synthesize the evidence. This meta-analytic study was conducted to assess the effect of high- and low-GI breakfast on subsequent short-term food intake. Trials were identified through MEDLINE, EMBASE, Web of Science, and Cochrane Central Register of Controlled trials, and manual searches of bibliographies until May 2015. Randomized controlled and cross-over trials comparing the effect of low- with high-GI breakfast on subsequent energy intake among healthy people were included. Nine studies consisting of 11 trials met the inclusion criteria. Only one trial was classified with high methodological quality. A total of 183 participants were involved in the trials. The meta-analytic results revealed no difference in breakfast GI (high-GI *vs.* low-GI) on subsequent short-term energy intake. In conclusion, it seems that breakfast GI has no effect on short-term energy intake among healthy people. However, high quality studies are still warranted to provide more concrete evidence.

## 1. Introduction

The glycemic index (GI), first introduced in 1981 [1], is a physiological assessment of the quality of carbohydrate (CHO)-rich foods in terms of their post-prandial glycemia *in vivo* in human. The GI is defined as the incremental area under the blood glucose response curve (iAUC) after a portion of food containing 50 g of available carbohydrate (aCHO), expressed as a percentage of that after the same amount of CHO from a reference food, usually glucose or white bread, taken by the same subject. The GI value of high-GI foods is over 70, and that of low-GI foods is less than 55 [2]. Generally, low-GI foods are regarded as being digested and absorbed slowly, while high-GI foods are believed to be rapidly digested and absorbed, resulting in different glycemic responses [3]. Compared with low-GI foods, high-GI foods may elicit hyperglycemia and hyperinsulinemia during early postprandial period and subsequent dynamic fall to even hypoglycemic levels. Recent evidence suggests that the general curve shape may be similar between low- and high-GI foods, although the highest glucose peaks and iAUC values are different between them [4]. This physiological response may induce many hormonal and metabolic changes that may affect health and disease parameters. The benefits of low-GI foods are advocated for diabetes mellitus [5], cancer [6], sport performance [7], cardiovascular disease [8], appetite control [9,10], and weight reduction [11].

The traditional glucostatic theory [12] has hypothesized that there is causal relationship between glycemia and short-term appetite regulation, and one’s appetite is stimulated when glycemia drops below a “static” level. This theory has been supported by recent studies [13,14,15], showing that transient and dynamic fall of glycemia can signal sense of appetite and meal initiation. These findings imply that short-term appetite control may be affected by the source of energy such as the GI of foods [9,10]. Two opposite opinions regarding the effect of GI on short-term appetite have been proposed previously [16,17]. This debate seems to be partially addressed by a systematic review study [9], in which the authors concluded that low-GI foods may suppress short-term appetite, compared with high-GI foods. However, a meta-analysis was not conducted in this study. Additionally, this systematic review was published in 2007. Therefore, an updated systematic review and meta-analysis is warranted to further clarify the effect of GI on short-term appetite control. The present meta-analytic study aims to quantify the effect of GI of breakfast (*i.e.*, low-GI *vs.* high-GI) on short-term appetite regulation as measured by energy intake among healthy people.

In the present study, high-GI and low-GI are classified according to the reported GI value [2]. However, given that most studies did not report the GI value, we also classified breakfast as high-GI and low-GI if significant difference was found in the iAUC value between the treatments. Considering the effect of aCHO amount on the overall glycemic responses, the glycemic load (GL) was introduced previously [18,19]. To control the influence of GL, only the trials with similar aCHO amount were included in the present meta-analysis. Additionally, as energy and macronutrient intakes are the main confounding factors in affecting GI of foods [9], only trials with similar energy and macronutrients were included in our research. To avoid the influence of so-called “second-meal effect” [20], only trials after an overnight fast were included.

## 2. Methods

A published review protocol for the current meta-analysis was not available. The Preferred Reporting Items for Systematic Reviews and Meta-Analysis guideline (PRISMA [21]) was followed when carrying out the current research.

### 2.1. Eligibility Criteria

The articles included in this review meet the following inclusion criteria: (i) the study subject must be related to compare the effect of breakfast GI (*i.e.*, low-GI *vs.* high-GI) on energy intake at subsequent meal, and breakfast was defined as food or meal consumed after an overnight fast; (ii) the compared breakfast had similar energy and macronutrients content; (iii) used healthy humans as participants; (iv) applied randomized controlled or cross-over trials with short duration (*i.e.*, ≤1 day [17,22]); (v) reported GI values and/or significant difference in iAUC value between treatments; (vi) used food or meal in breakfast as the only treatment; (vii) outcome measures must be related to appetite and evaluated through objective approaches (*i.e.*, the food or energy intake in subsequent meals); and (viii) provided adequate information for effect size calculation. The studies were excluded if: (i) the study was not related to the effect of breakfast GI on energy intake at subsequent meal; (ii) animal models or unhealthy participants (e.g., obese and diabetic participants) were used; (iii) the study design were not randomized controlled or cross-over trials; (iv) the duration of the trial was long (*i.e.*, >1 day); (v) used other treatments rather than only food or meal, e.g., exercise; (vi) employed subjective measures to evaluate appetite; (vii) lack of information for calculating the effect size, even when efforts had been made to obtain the relevant data from the correspondences; (viii) they were abstracts; and (ix) they were published in non-English journals.

### 2.2. Search

We searched studies from four major electronic databases from inception until 27 May 2015: MEDLINE (1946–), EMBASE (1947–), Web of Science (1900–), and Cochrane Central Register of Controlled Trials (1898–). The following two groups of keywords were combined for the search: (i) “glycemic index” OR “glycemic indices” OR “glycemic index number *” OR “glycaemic index” OR “glycaemic indices” OR “glycaemic index number *”; AND (ii) “appetite *” OR “appetite regulation” OR “hunger” OR “satiety” OR “satiation”. In addition to the database search, the reference lists of the identified articles and relevant review articles [9,17] were also manually searched. Finally, a number of experts from the field of GI were contacted to find additional studies.

### 2.3. Study Selection

Two authors (F.S. and C.L.) conducted the literature search and removed the duplicates. Two reviewers (F.S. and Y.Z.) independently screened the eligible articles from the initial search by reading through the titles and abstracts. Full texts were further sought if the titles or abstracts did not provide enough information to decide whether the study should be included or excluded. Advice was sought from the third reviewer (C.L.) to reach a consensus when disparity on the inclusion or exclusion of an article occurred between the two reviewers.

### 2.4. Data Collection Process and Items

Two authors (F.S. and C.L.) independently extracted the following data from the included studies: (i) author and year of publication; (ii) characteristics of the participants: sample size, sex, and age; (iii) study design; (iv) treatment or test food/meal; and (v) effect size (ES: *i.e.*, standardized mean difference) or raw data for ES calculation. No disparities of data abstraction between the two authors were observed.

### 2.5. Risk of Bias in Individual Studies

Two authors (F.S. and Y.Z.) independently assessed the risk of bias of the included studies using the Jadad scale [23]. The scale has been widely used in assessing methodological quality in the nutritional field [24,25]. The scale has three components: randomization (2 points), blinding (2 points) and reported withdrawals (1 point). The Jadad score ranges from 0 to 5. A study with a score higher than 3 is considered to have high quality. Disagreements were solved after discussion between the two authors.

### 2.6. Synthesis of Results

Meta-analysis was conducted through Comprehensive Meta-Analysis Software. The Q-test was used to examine whether the pooled ES vary between studies. A statistical significant Q-value indicates differences of study heterogeneity. The *I*^2^ statistic was calculated to measure the effect of heterogeneity. The values for *I*^2^ at 25%, 50%, and 75% indicates low, moderate, and high heterogeneity, respectively [26]. If substantial heterogeneity was not found (*i.e.*, a non-significant Q-test result and/or an *I*^2^ statistic smaller than 50%), a meta-analysis with a fixed-effects model was conducted to pool ESs with 95% confidence interval (CI); otherwise, a random-effects model was employed [27]. Finally, the Egger regression asymmetry test and Begg’s funnel plot were applied to assess publication bias.

## 3. Results

### 3.1. Study Selection

**Figure 1:**
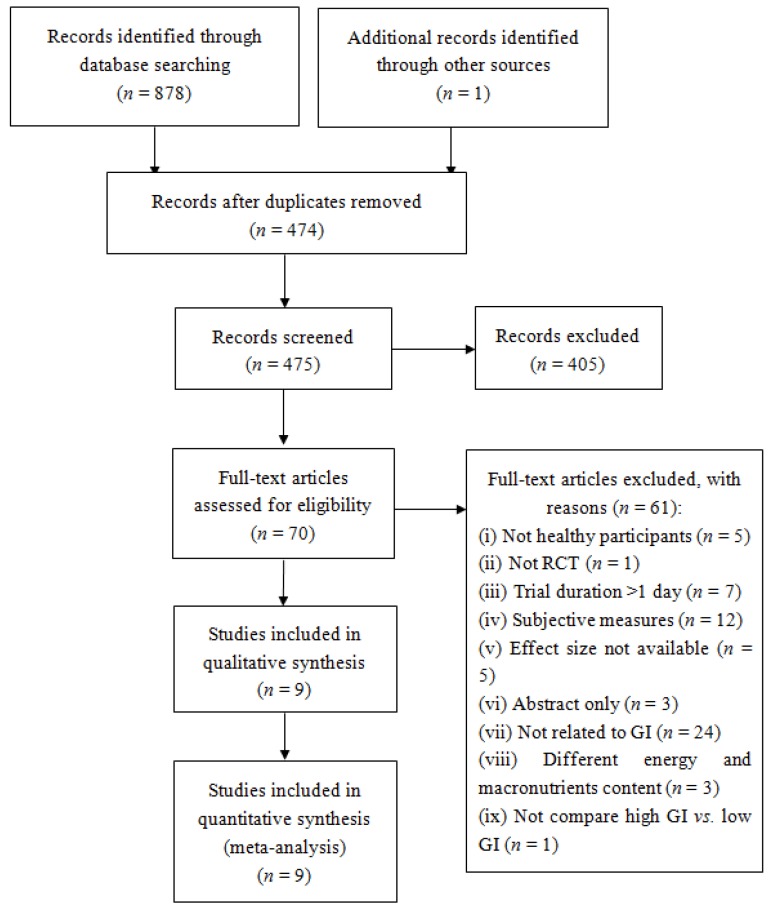
Flow diagram of study selection process.

The flow of the study selection is shown in Figure 1. A total of 879 papers were searched. After removing duplications (*n* = 474), 405 articles were excluded after reading the titles and abstracts. The remaining articles (*n* = 70) were further assessed for eligibility based on the full texts. Finally, nine studies consisting of 11 trials (two studies had two eligible trials) were included in this review [28,29,30,31,32,33,34,35,36,37].

### 3.2. Study Characteristics

The main characteristics of included studies are summarized in Table 1. The included studies were published between 1991 and 2012 (two studies were published before 2000). A total of 183 participants were involved in the 11 trials. The sample size for these trials ranged from six to 28. Most participants were male adults (Male = 128, Female = 55). The test meal (breakfast) varied a lot among the trials. As described, GI and/or iAUC/AUC values were used to classify low- and high-GI breakfast. Most trials (*n* = 10) reported iAUC/AUC values. The interval between breakfast and subsequent energy intake ranged from 30 to 180 min. In terms of subsequent energy intake (*ad libitum* meals), different types of food were provided, with pizza being the most popular among the trials (*n* = 5).

### 3.3. Risk of Bias within Studies

All the included trials had relatively high risk of bias. In particular, none of the included trials used double blinding. Although all the included trials used a randomization design, most (*n* = 10) failed to report randomization approaches. Moreover, only four trials explained the number of withdrawals or reasons for dropout (Table 2).

**Table 1:** Characteristics of eligible studies included in this meta-analysis.

First Author and Year	Participants	Design	Criteria for GI Level (Duration)	Test Meal		Subsequent Food Intake	Key Findings
				Low-GI	High-GI		
Anderson (2002) [28], Trial 2	*n* = 18 Age: 20–30 Gender: 18 M	RCT	AUC (60 min)	Amylose (75 g aCHO, 0 g protein, 0 g fat, 0 g fiber, 300 kcal) beverage	Amylopectin (75 g aCHO, 0 g protein, 0 g fat, 0 g fiber, 300 kcal); Sucrose (75 g aCHO, 0 g protein, 0 g fat, 0 g fiber, 300 kcal); Polycose (75 g aCHO, 0 g protein, 0 g fat, 0 g fiber, 300 kcal) beverage	Pizza meal according to one’s preference served at 60 min	Polycose resulted in significantly less food intake than did amylopectin
Anderson (2002) [28], Trial 3	*n* = 18 Age: 18–35 Gender: 18 M	RCT	AUC (60 min)	Fructose-glucose (75 g aCHO, 0 g protein, 0 g fat, 0 g fiber, 300 kcal) beverage	Sucrose (75 g aCHO, 0 g protein, 0 g fat, 0 g fiber, 300 kcal); Polycose (75 g aCHO, 0 g protein, 0 g fat, 0 g fiber, 300 kcal); Glucose (75 g aCHO, 0 g protein, 0 g fat, 0 g fiber, 300 kcal) beverage	Pizza meal according to one’s preference served at 60 min	No difference
Anderson (2010) [29], Trial 1	*n* = 17 Age: 20–30 Gender: 17 M	RCT	AUC (30 min)	Regular cornstarch with tomato soup (a high-amylopectin granular starch, 46.5 g aCHO, 1 g protein, 0 g fat, <1.5 g fiber, 190 kcal)	Malto-dextrin with tomato soup (a highly processed, non-granular starch, 47 g aCHO, 1 g protein, 0 g fat, 1 g fiber, 192 kcal)	Pizza meal according to one’s preference served at 30 min	No difference
Anderson (2010) [29], Trial 2	*n* = 16 Age: 20–30 Gender: 16 M	RCT	AUC (120 min)	Regular cornstarch with tomato soup (46.5 g aCHO, 1 g protein, 0 g fat, <1.5 g fiber, 190 kcal)	Malto-dextrin with tomato soup (47 aCHO, 1 g protein, 0 g fat, 1 g fiber, 192 kcal)	Pizza meal according to one’s preference served at 120 min	No difference
Flint (2006) [30]	*n* = 28 Age: 24.8 (0.5) Gender: 28 M	RCT	iAUC (180 min)	Reference bread (50 g aCHO, 12 g protein, 7 g fat, 5 g fiber, 319 kcal); Frosties + milk (50 g aCHO, 9 g protein, 3 g fat, 1 g fiber, 270 kcal)	Porridge + rolled oats + water + applesauce (50 g aCHO, 8 g protein, 5 g fat, 6 g fiber, 289 kcal)	A pasta salad served at 180 min	No difference
Furchner-Evanson (2010) [31]	*n* = 21 Age: 20–30 Gender: 21 F	RCT	AUC (120 min)	Dried plums (48 g aCHO, 3 g protein, 0 g fat, 6 g fiber, 238 kcal)	Low-fat cookies (54 g aCHO, 4 g protein, 0 g fat, 0 g fiber, 238 kcal); White bread (42 g aCHO, 6 g protein, 3 g fat, 3 g fiber, 238 kcal)	A meal (strawberry flavored low-fat yogurt and granola) served at 120 min	No difference
Holt (1995) [33]	*n* = 9 Age: 19.3–29.0 Gender: 4 M, 5 F	RCT	AUC (120 min)	Ordinary boilded rice (50 g aCHO, 4.2 g protein, 0.4 g fat, 1.5 g fiber, 218 kcal); High amylose puffed rice cakes (50 g aCHO, 6.3 g protein, 2.1 g fat, fiber 2.7 g, 235 kcal)	Quick-cooking rice (50 g aCHO, 5.4 g protein, 0.6 g fat, 1.1 g fiber, 214 kcal); Low amylose puffed rice cakes (50 g aCHO, 4.8 g protein, 2.0 g fat, 2.6 g fiber, 228 kcal)	Eat freely from a limited range of food items served at 120 min	No difference
Kaplan (2002) [34]	*n* = 20 Age: 60–82 Gender: 10 M, 10 F	RCT	AUC (105 min) + GI value (white bread as the reference)	Pearled barley (46.6 aCHO, 5.9 g protein, 2.7 g fat, 9.4 g fiber, GI = 36, 228 kcal)	Instant mashed potato (49.5 g aCHO, 5.1 g protein, 2.2 g fat, 3.1 g fiber, GI = 118, 233 kcal)	Lunch (sandwiches, muffins, cookies) served at 120 min	No difference
Kristensen (2010) [35]	*n* = 16 Age: 24.1 (3.8) Gender: 6 M, 10 F	RCT	GI value (white bread as the reference)	Refined wheat pasta (50 g aCHO, 24 g protein, 17 g fat, 2.2 g fiber, 454 kcal, GI = 38)	Refined wheat bread (50 g aCHO, 23 g protein, 17 g fat, 3.6 g fiber, 444 kcal, GI = 100)	Pizza meal served at 180 min	No difference
Lumaga (2012) [36]	*n* = 14 Age: 24–39 Gender: 8 M, 6 F	RCT	AUC (180 min)	Control beverage (37.3 g aCHO, 0 g Protein, 0 g fat, 0 g fiber, 149 kcal)	Fruit-based beverage (34.3 g aCHO, 1.0 g protein, 0.3 g fat, 2.5 g fiber, 149 kcal)	Compose lunch tray based on one’s own desire to eat served at 180 min	No difference
Rodin (1991) [37]	*n* = 6 Age: 35.6 (2.4) Gender: 3 M, 3 F	RCT	AUC value (135 min)	Pudding sweetened with fructose (50 g aCHO, 24% protein, 41% fat, 530 kcal)	Pudding sweetened with glucose (50 g aCHO, 24% protein, 41% fat, 520 kcal)	Buffet lunch served at 135 min	Lower energy intake after low-GI food intake

**Table 2:** Methodological quality of included trials.

First Author and Year	Randomization	Double Blinding	Withdrawals	Appropriate Randomization	Appropriate Double Blinding	Total
Anderson (2002) [28], Trial 2	1	0	0	0	0	1
Anderson (2002) 28], Trial 3	1	0	1	0	0	2
Anderson (2010) [29], Trial 1	1	0	0	0	0	1
Anderson (2010) [29], Trial 2	1	0	0	0	0	1
Flint (2006) [30]	1	0	0	1	0	2
Furchner-Evanson (2010) [31]	1	0	1	0	0	2
Holt (1995) [33]	1	0	0	0	0	1
Kaplan (2002) [34]	1	0	1	0	0	2
Kristensen (2010) [35]	1	0	1	0	0	2
Lumaga (2012) [36]	1	0	0	0	0	1
Rodin (1991) [37]	1	0	0	0	0	1

### 3.4. Synthesis of Results

**Figure 2:**
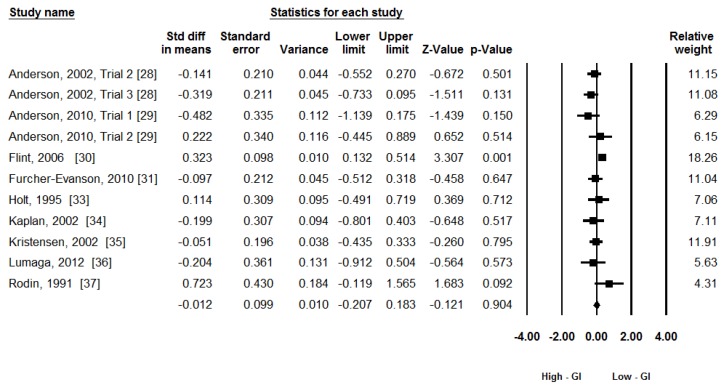
The effects of low-GI and high-GI breakfast on subsequent energy intake. (GI: Glycemic Index).

Energy intake (kJ) was used as the principal outcome measure in this meta-analysis with 11 trials. A random-effects model was used given the moderate heterogeneity: Q_(10)_ = 18.70, *p* = 0.04, *I*^2^ = 46%. Figure 2 shows the forest plot of the analysis. The meta-analytic results didn’t show any effect of GI on subsequent energy intake: ES = −0.01, 95% CI (−0.21, 0.18), z = −0.12, *p* = 0.90.

### 3.5. Publication Bias

**Figure 3:**
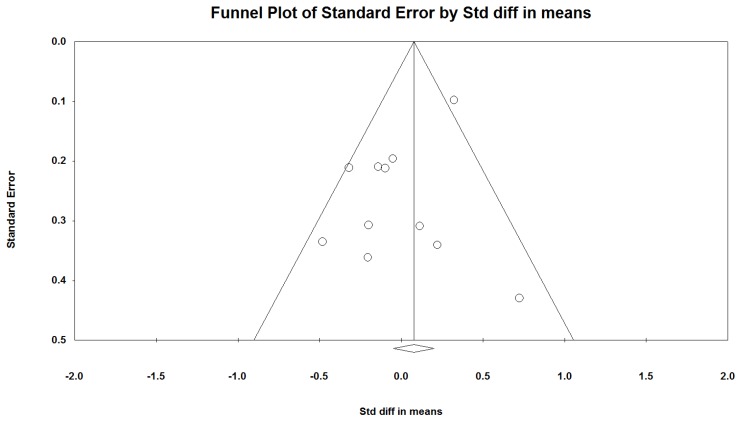
Funnel plot.

The non-significant results of Egger’s test indicated no publication bias (*p* = 0.46). However, the funnel plot was not symmetrical (Figure 3). The unsymmetrical plot may be due to the heterogeneity among the included studies rather than publication bias.

### 3.6. Additional Analysis

**Figure 4:**
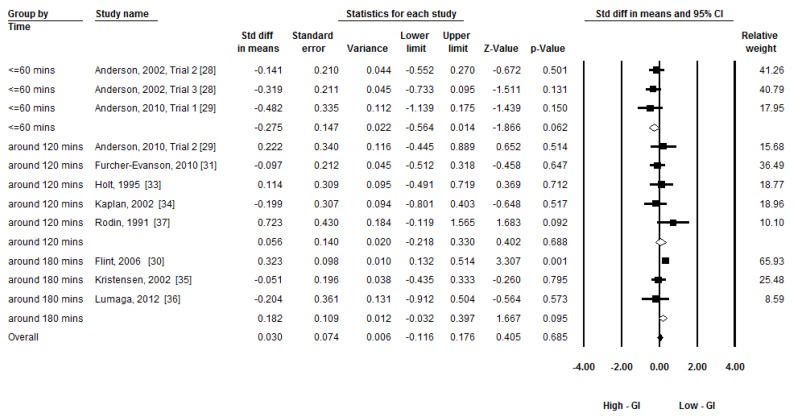
Subgroup meta-analysis: The effect of low-GI and high-GI breakfast on subsequent energy intake at 60, 120, and 180 min. (GI: Glycemic Index).

**Figure 5:**
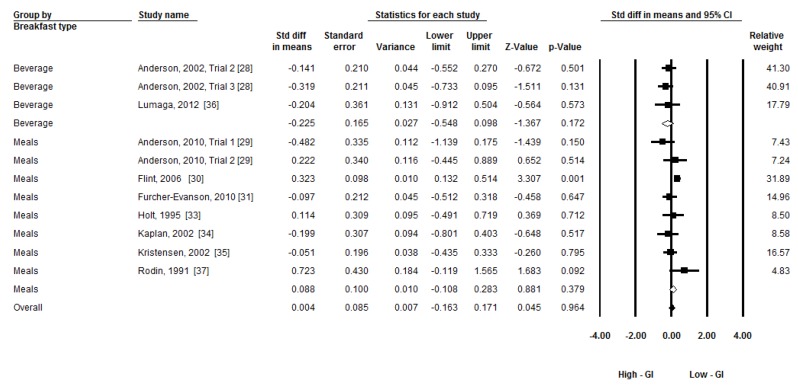
Subgroup meta-analysis: The effect low-GI and high-GI breakfast with different types on subsequent energy intake. (GI: Glycemic Index).

**Figure 6:**
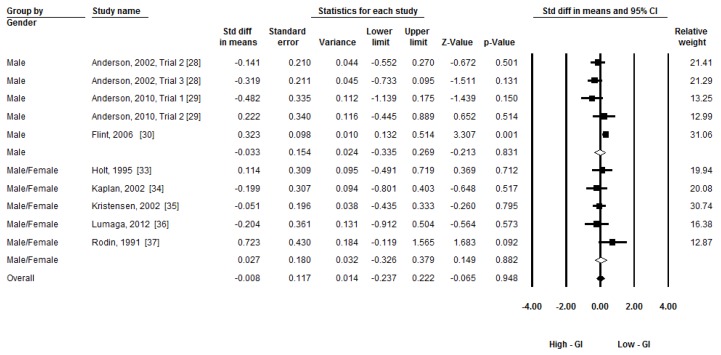
Subgroup meta-analysis: The effect of low-GI and high-GI breakfast on subsequent energy intake between gender groups. (GI: Glycemic Index).

Given the moderate heterogeneity, three subgroup analyses were conducted to compare the group differences by time of subsequent food/meal intake (less than 60 min, around 120 min, and around 180 min), types of breakfast (beverage, food/meal other than beverage), and gender (male, both). A random-effects model was applied to conduct the subgroup analyses [27]. Figure 4, Figure 5 and Figure 6 present the forest plots of the subgroup analyses. There was a group difference on subsequent energy intake in time (Q_(2)_ = 6.26, *p* = 0.04). There was a trend that breakfast GI had an effect on subsequent food intake within 60 min (ES = −0.28, *p* = 0.06). No group differences in types of breakfast (Q_(1)_ = 2.64, *p* = 0.10) and gender (Q_(1)_ = 0.06, *p* = 0.80) were observed.

## 4. Discussion

This meta-analytic study was conducted to examine the effect of different GI breakfast (low-GI *vs.* high-GI) on subsequent short-term energy intake among healthy people. The major finding of this research was that, based on the pooled ESs of 11 randomized cross-over trials, different GI breakfast did not show any effect on subsequent short-term energy intake. This finding is inconsistent with an early systematic review [9], in which low-GI foods was suggested to suppress short-term appetite compared with high-GI foods. The different findings may be due to the varying research scopes. For example, the current study focused on the effect of breakfast rather than other meals, such as lunch or dinner. In addition, the previous systematic review used participants with different health conditions (e.g., obese, diabetes). The current review was confined to healthy participants only. Thus, the study characteristics of the current review are more homogenous than those of the previous systematic review. More importantly, in the previous systematic review [9], no meta-analysis was conducted, and the conclusion was largely based on a simple counting of positive results. Therefore, the current review provided the first piece of evidence on the effect of breakfast GI on subsequent energy intake through meta-analysis.

According to our meta-analytic results, it is still premature to conclude that breakfast GI affects short-term energy intake among healthy individuals. According to the previous systematic review [9], by using a subjective assessment method, most of the included studies supported an increase in satiety after low-GI *versus* high-GI food/meal consumption. As studies with subjective measurement are not included in the present review, it is hard to make the direct comparison between the two studies. However, according to the results of these two studies, it is possible that subjective appetite feelings are not directly linked to energy intake. It should also be noted that most of the included studies have low research quality (Table 2). A few limitations should be accounted while interpreting the current findings. Similar to other meta-analytic studies, only articles published in English were included. The studies that did not report GI values or iAUC/AUC results were excluded from this meta-analysis. Additionally, although we have tried to exclude the potential influences of confounding factors, it is very difficult to control all of them. Small differences in fiber contents (~5 g) still exist between two trials in some studies [30,34]. Viscosity of beverages may also affect gastrointestinal hormonal responses and appetite [38], which was not controlled in the present systematic review. Because of these limitations, the research findings may be biased. More high-quality studies are warranted, especially those with strictly controlled energy, macronutrients, fiber, viscosity, and with higher Jadad scores.

Three subgroup meta-analytic analyses were conducted in our research to investigate the possible explanations of heterogeneity. The postprandial time (≤60 min, around 120 min, around 180 min) after breakfast consumption and types of breakfast (beverage *vs.* breakfast other than beverage) showed no effect on subsequent energy intake. However, the results of the present study show that food intake time (≤60 min) tends to affect the effect of GI on subsequent energy intake (*p* = 0.06). Glucostatic theory [12,13] may be one possible reason to explain the effect of GI on subsequent energy intake. It indicates that transient glycemia drops after high-GI food consumption may stimulate appetite and may be responsible for short-term appetite regulation. By contrast, low-GI food consumption blunts the hyperglycemia and hyperinsulinemia, which may reduce the subsequent fall in glycemia and prevented reactive hypoglycemia. The sustained exogenous supply of blood glucose may be caused by slower and lengthened digestion and absorption of low-GI food. However, this theory only accounts for the difference in effect of GI on food intake for several, but not all of previous studies [9]. One previous study also suggests that insulin itself does not affect short-term feeding behavior [39]. Furthermore, recent evidence shows that slowly digestible starch may not always result in reduced postprandial glycemia because of a slower glucose clearance rate [40]. According to Brand-Miller [4], the peak glucose concentration occurs at around 30 min after high- or low-GI food/meal consumption. Therefore, it is possible that the most significant difference in blood glucose concentrations during the postprandial period will be observed within 60 min. However, the lowest blood glucose concentrations were observed at around 120 min in both trials. Therefore, the different glycemic responses after high- or low-GI food consumption are expected at different postprandial periods. This may affect energy intake to a certain degree. However, it cannot be explained by glucostatic theory.

The subgroup analyses indicated that breakfast GI has no effect on subsequent energy intake in males and in both genders (Figure 6). Among all the included studies, only one study used female participants [31]. Therefore, the study (female group) is not included in the sub-group analysis regarding gender. Although several studies have included both male and female participants [32,33,34,35,36,37], the results of both genders have been combined for further analysis, which may partly be attributed to the fact that both males and females were considered to be equal in GI measurement [3]. However, recent evidence suggests that females may be more sensitive to macronutrients and overfeeding, which may result in different subsequent energy intake between genders [41,42]. This gender difference may be caused by estrogens in females [43]. Therefore, it is possible that breakfast with different GI has different effect on subsequent short-term energy intake among healthy females. Furthermore, another two excluded studies [44,45] using female participants found that low-GI beverage/food may suppress subsequent energy intake compared with high-GI beverage/food, although different macronutrients between two trials in these two studies may affect the final results. Therefore, because of limited number of included studies, further studies are still needed to analyze the research findings by gender groups.

Except for the glucostatic theory [12], several other possible mechanisms may also mediate appetite regulation after different GI meal intake. First, the gut–brain axis is the key component in the recently established model of appetite regulation [46]. Various peptide hormones are secreted in the gastrointestinal tract that may regulate appetite, including ghrelin, cholecystokinin, glucagon-like-peptide-1, and peptide-YY [47]. The satietogenic effect of low-GI foods over high-GI foods was hypothesized for their slower digestion and prolonged presence in the gastrointestinal tract, and thus stimulation of gut satiety signals [48]. Second, low-GI starchy foods were associated with an increased amount of CHO escaping digestion in the small intestine [49,50], and hence an increase in colonic fermentation [51,52]. Dietary fibers, resistant starch, and non-digestible oligosaccharides are unavailable CHO to humans *in vivo* [53]. One recent review suggested the possible mechanism of the appetite suppressing effect mediated by colonic fermentation of unavailable CHO in low-GI foods [54]. Third, brain activities may be involved in appetite regulation. One recent study found that high-GI meal consumption may selectively stimulate the brain regions associated with reward and craving four hours after meal consumption, compared with iso-caloric low-GI meal intake [55]. Although these different mechanisms have been proposed for appetite regulation after low- and high-GI meal consumption, they may only account for a part of the previous studies. According to the results of the present review, more carefully designed studies are still needed to further clarify the effect of GI on appetite, especially considering the gender difference and potential mechanisms.

The practical utility of GI for long-term appetite control and weight management were more complicated because of the truthfulness of predicted GI [56], least significant difference of GI [57], limitations of dietary recommendations and counseling [58], as well as redundant homeostatic mechanisms to offset negative energy balance [59]. These questions cannot be answered by the present review.

## 5. Conclusions

In conclusion, it seems that breakfast GI has no effect on short-term energy intake among healthy people. However, this conclusion should be interpreted with caution given that most of the included trials are of low research quality, and some confounding factors are not well controlled.
